# Successful embolization of giant pulmonary artery pseudoaneurysm using coils and ethylene vinyl alcohol copolymer (Onyx)

**DOI:** 10.1016/j.radcr.2021.02.029

**Published:** 2021-02-24

**Authors:** Noémie Lutz, Sylvain Grange, Christian Sanson, Rémi Grange

**Affiliations:** a Department of Radiology, University Hospital of Saint-Etienne, France; b Department of Pneumology, University Hospital of Saint-Etienne, France

**Keywords:** Lung, Embolization, Hemoptysis, Pulmonary artery pseudoaneurysm

## Abstract

Hemoptysis could be a life-threatening event, especially when the bleeding originates from the arterial pulmonary circulation. The main cause of this type of bleeding is pulmonary artery pseudoaneurysm (PAP), which can be managed by surgical, medical or minimally invasive techniques. This study reports the case of massive hemoptysis in a 75-year-old male patient, with a former history of lobectomy. The initial CT scan showed a giant PAP from a branch of the right middle lobar pulmonary artery, within the right lower lobectomy cavity. An endovascular approach was decided. Subsequently, the feeding artery of the PAP was embolized with detachable coils. The control CT scan showed a persistent opacification of the PAP. The embolization was then completed by injection of Onyx within coils packing, with a complete thrombose of the PAP on control CT scan. This report confirms the safety and efficacy profile of an endovascular approach to treat giant PAP, using a combination of coils and Onyx.

## Introduction

Hemoptysis is defined as the exteriorization of red aerated blood from the mouth following a cough episode. Massive hemoptysis (95%) usually originates from bronchial circulation [1]. Less than 10% of all cases originate from pulmonary artery circulation. Cases originating from pulmonary artery circulation require a specific diagnosis and therapeutic approach. The most common etiology of bleeding from pulmonary artery circulation is pulmonary artery pseudoaneurysm (PAP) due to erosive inflammatory process. Traumatic PAPs represent a very rare cause of hemoptysis. In this case report, we describe the case of a patient with giant PAP due to bronchial fistula, which occurred 7 years after lobectomy for adenocarcinoma, and was successfully treated by combination of coils and Onyx.

## Case description

A 75-year-old patient was admitted to the hospital for massive hemoptysis and fever. The patient has a relevant history of lung adenocarcinoma which had begun 7 years prior to admittance to the hospital and was treated by neo-adjuvant chemotherapy (Cisplatin and Pemetrexed), a right lower lobectomy, and adjuvant radiotherapy. He had complete remission following these treatments. A bronchial fibroscopy performed during follow-up found hemoptoic secretions without active bleeding and a bubbling suggesting a fistula, without suspicious lesion or purulent secretion.

**Fig. 1:**
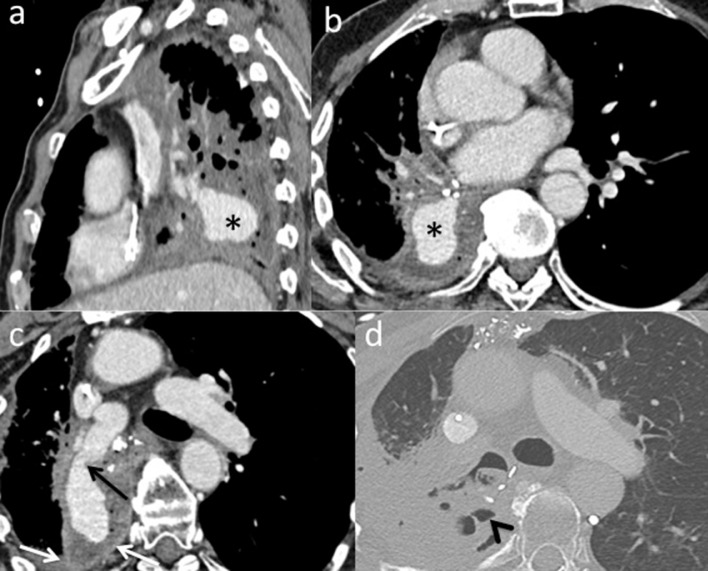
Chest computed tomography (CT) scans with arterial time injection in sagittal, axial section, in the long axe of the aneurysm sac (a-c), in mediastinal window and axial section in parenchymal window (d) show contrast media filling of the aneurysm sac in the right lower lobectomy cavity (*), feeding from the middle pulmonary artery (black arrow). Contrast-enhancement (white arrows) and air (arrow's head) within the lobectomy cavity suggest an infection.

**Fig. 2:**
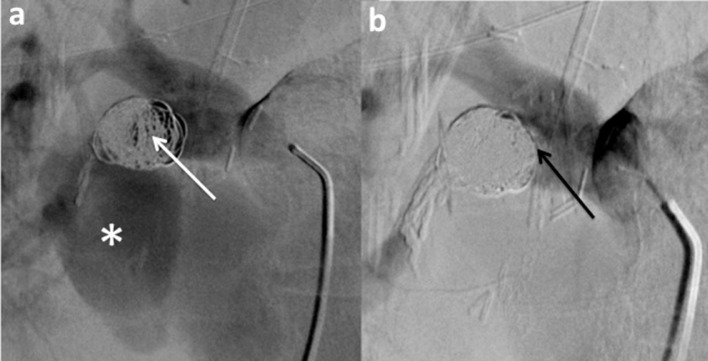
Angiographic opacification of the right pulmonary artery at the beginning of the second procedure (a) shows persistent filling of the coil packing (white arrow) and the aneurysmal sac (*) by iodine contrast. Angiographic opacification after adjunction of Onyx (b) shows no more filling of coil packing (black arrow) and the aneurysmal sac.

**Fig. 3:**
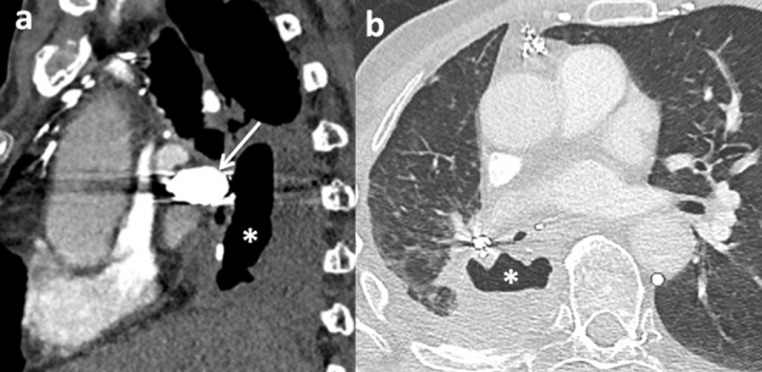
Chest computed tomography (CT) scans with arterial time of injection, 1 month after the second procedure, (sagittal section in mediastinal window (a) and axial section in parenchymal window (b)), show a regression in size and liquid component of the cavity (*), with coil packing of the aneurysmal neck (white arrow).

On clinical examination, he showed acute dyspnea, an acute anemia (hemoglobin level of 9g/dL), and initial hemodynamic instability, which required 1.5 mg/h norepinephrine. An emergency CT-scan revealed a large pseudoaneurysm from a branch of the right middle lobar pulmonary artery, within the right lower lobectomy cavity, measuring 41 × 22 × 33 mm, with a 9 mm neck (Fig. 1). After a multidisciplinary meeting, an endovascular approach was decided. A 6 French (Fr.) sheath (Terumo, Tokyo, Japan) was inserted into the right femoral vein. Catheterization of the feeding artery was performed using a hydrophilic 0.035-inch guidewire, a 5-F Vertebral catheter (Terumo, Tokyo, Japan), and a 2.8-F microcatheter (Terumo, Tokyo, Japan). The feeding artery was embolized using 3 detachable 3D-coils of 11 mm × 28 cm, 12 mm × 31 cm and 16 mm × 39 cm Concerto (Medtronic, Dublin, Ireland) and 5 detachable fibered coils (4 coils 6 mm × 10 cm and one coil 6 mm × 10 cm) Interlock (Boston Scientific, Marlborough, MA, USA). The patient presented a persistent hemoptysis 4 days after the procedure. Control chest CT-scan showed a persistent permeability of the pseudoaneurysm. The embolization was then completed by the adjunction of 3.6 mL of Onyx (Medtronic, Dublin, Ireland) within coil packing, by right femoral access, using a Transcend 0.35 guidewire (Boston Scientific, MA, USA), a 5F Vertebral catheter and Headway 21 microcatheter (MicroVention, Aliso Viejo, CA, USA). The angiographic control did not show any filling of the PAP (Fig. 2). The patient did not show a new hemoptysis event. During hospitalization, a blood culture was positive for *Staphylococcus aureus*, suggesting endocarditis of the tricuspid valve, which was not confirmed by a trans-thoracic echography. He was discharged 3 weeks after the second procedure. A follow-up computed tomography one month after the second procedure showed a complete thrombosis of the aneurysm sac and a size reduction of the lobectomy cavity and a reduction of the liquid component (Fig. 3).

## Discussion

To our knowledge, this report is the first to describe a delayed occurrence of PAP in the context of necrotic infection of lobectomy cavity with fistula and which was treated by an endovascular approach. It remains to be determined if the infection favored the fistula or if the fistula was the source of the necrotizing infection, which resulted in the formation of the pseudoaneurysm.

PAP is a focal dilation of a pulmonary artery segment, involving the adventice and a higher risk of rupture than a true aneurysm. It is a rare pathology and is underdiagnosed by radiologists. However, this pathology can be life-threatening if undiagnosed, with a high mortality rate (50%). In case of abundant hemoptysis, a CT scan is the gold standard. It must follow a specific protocol [2] to eliminate pulmonary arterial origin, locate the bleeding site and establish the cause. PAP appears as an irregular artery or an image of addition close to a necrotic zone.

Historically, the main cause of PAP was tuberculosis, first described by Rasmussen. It results from the contiguous propagation of the pathogen, which leads to tissue destruction of the outer vessel wall. Prevalence of PAP decreased after the discovery and widespread use of antibiotics, especially in Western countries. More recently, the main infectious causes of PAPs are represented by tricuspid endocarditis and necrotizing pneumonia. Neoplasm is the second cause of symptomatic PAPs [3], which can result in tumoral invasion of the vascular wall. Traumatic causes are less common, mainly represented by arterial catheterization and penetrating wounds.

Chen et al [3] described 35 PAPs in 24 patients, but 13 of 24 (54.1%) patients had hemoptysis. The main cause of symptomatic PAP was infection (46.1%) and neoplasm (15.4%). Only one 73-year-old patient presented with hemoptysis 3 weeks after undergoing lobectomy (without fistula), with a solitary symptomatic PAP was treated by use of covered stent.

Management of PAP includes medical treatment, surgical management [4] and minimally invasive techniques. Surgical treatment is associated with a high risk of morbidity and mortality and should be reserved for patients with minimal invasive treatment failure. Minimal invasive techniques, including endovascular or percutaneous approaches under CT-scan [5] have become a widespread initial therapy for these patients. Various embolizing agents have been described either by treating the aneurysmal sac or neck, such as coils [3], liquid agents (N-butyl cryanoacrylate, thrombin, Onyx), and stents [3]. The choice depends on the size of the aneurysm, the size of the neck, the location (proximal or distal), and the experience and habits of the interventional radiologists [6]. For small pseudoaneurysms, a simple coiling may be sufficient, but expose to the risk of rupture during filling of the aneurysm sac. Use of covered stent maintains permeability of the feeding artery but exposes to risks of thrombosis or migration. Liquid agents are frequently used [5,7], because of a lower risk of aneurysm rupture, and its ability to occlude the aneurysm neck, but expose to nontarget embolization. When the endovascular approach is not possible, percutaneous embolization with injection of thrombin or glue, under CT scan, fluoroscopy or ultrasound guidance, may be technically easier in case of peripheral location [5]. In our case, the size of the neck and the high flow within the pulmonary artery explains the persistent filling of the aneurysm after coils packing. The combination of coils and liquid agent has previously shown its effectiveness in pulmonary pseudoaneurysms [7]. The coils are then used to protect the glue reflux, and the glue to increase the thrombogenic effect of the coils . The large size of the aneurysm and the nonfunctionality of the right lower lobe led us to choose to occlude the feeding artery of the pseudoaneurysm. The risk of reflux and nontarget embolization within functional branches of the right pulmonary artery motivated us to prefer Onyx from N-butyl cyanoacrylate.

This report confirms that the use of a combination of coils and Onyx can represent a successful approach for hemoptysis treatment of giant PAP bleeding. A multidisciplinary approach of this rare pathology is essential, allowing a treatment adapted to the etiology and the characteristics of the aneurysm, and the clinical state of the patient.

## Consent

I, Dr. R. GRANGE, confirm that consent was requested from the patient prior to the completion of the article.
